# Editorial: Selenium and human health

**DOI:** 10.3389/fnut.2023.1269204

**Published:** 2023-09-05

**Authors:** Xianli Gao

**Affiliations:** School of Food and Biological Engineering, Jiangsu University, Zhenjiang, China

**Keywords:** selenium, health, nutrition, bioactivity, toxicity

Selenium (Se) is an indispensible trace element to human health. Due to the uneven distribution of selenium around the world, it leads to insufficient or excessive Se intake among residents in some regions (1). The World Health Organization (WHO) recommends the intake of Se ranged from 50 to 200 μg/d/adult (2). Notably, the impact of Se on human health is not only related to the intake of Se, but also closely related to the type and valence of Se (3–5). Currently, it has been proved that more than 40 diseases are highly related to Se deficiency (6, 7), such as Keshan disease, cataract, diabetes, thyroid disease, cancer, etc. (Cai et al.). However, excessive Se intake can also lead to skin damage, nail and hair loss, gastrointestinal discomfort, neurological damage, paralysis, and even death (8). Therefore, Se plays a vital role in human health. In the face of issues such as insufficient or excessive Se intake caused by the uneven distribution of Se in geographical locations, it is of great significance to further understand the latest research advances in Se and human health.

The relationship between gut microbiota and health is an emerging research hotspot recently. Cai et al. reviewed the regulatory mechanism of Se on human gut microbiota and its impact on diseases. They concluded that insufficient Se intake might lead to changes in the phenotype of intestinal microbiota, making humans more vulnerable to inflammatory disease, thyroid disease, cancer, etc. Meanwhile, the authors also pointed out shortcomings of the current research and the future research directions: (i) relationship between the optimal daily Se intake of human beings and the Se demand of normal gut microbiota needs further research; (ii) more clinical studies are needed to prove the relationship between human gut microbiota and occurrence of diseases; (iii) the combination of high-throughput sequencing and multi-omics can solve the difficulties in the research of effect of Se on gut microbiota.

The long-term influence of serum Se level on the health of cardiovascular patients with chronic kidney disease is unclear till now. Zhu et al. from Sichuan University investigated the correlation between serum Se levels and all-cause /cardiovascular disease (CVD) mortality in 3,063 CVD patients with chronic kidney disease (CKD), and estimated the hazard ratios (HRs) and 95% CI using Cox professional hazards model and competitive risk model. Results indicated that 884 deaths occurred including 336 CVD-associated deaths, the median (IQR) concentration of serum Se of the deaths was 181.7 (156.1–201.5) μg/L. After Se supplement, the mortality of CVD patients with CKD including CVD and all-cause mortality decreased with the increase of serum Se level (*p* < 0.001). The multivariate-adjusted HRs (95% CI) were 0.513 (0.356–0.739) for CVD mortality (*p*_trend_ < 0.001) and 0.684 (0.549–0.852) for all-cause mortality (*p*_trend_ < 0.001) when Se levels were compared to the extreme quartiles, suggesting that higher serum Se level was associated with lower risk of CVD and all-cause mortality among CVD patients with CKD.

Chen X. et al. investigated the protective effect of different Se supplements on liver injury caused by heat stress and exercise fatigue and the related mechanisms. SD rats were continuously gavaged with Se-rich soy protein (SePro), Na_2_SeO_3_, common peptides (PPs), Se-rich soy peptides (SePPs) and selenomethionine (SeMet) for 7 days. Results showed that the levels of catalase, superoxide dismutase, malondialdehyde in serum and activities of GSH and GSH-Px in liver of SePPs rats were significantly different from those in the control group (*p* < 0.05), SePPs significantly increased the antioxidant level of SD rats, inhibited lipid peroxidation and reduced the activities of liver enzymes of SD rats. Histological studies found that the infiltration of inflammatory cells in the liver tissue of SePPs rats decreased and new cells appeared in livers of SePPs rats. In addition, SePPs increased glutathione content and GSH peroxidase activity in rat liver, and protected rat liver by regulating NF-κB/IκB pathway and preventing the release of interleukin-1 β (IL-1 β), interleukin-6 (IL-6) and tumor necrosis factor α (TNF-α). SePPs showed better liver protection effects than SePro, SeMet, Na_2_SeO_3_ and PPs. Therefore, SePPs can be used as a priority Se resource to develop supplement to prevent liver damage caused by heat stress and exercise fatigue.

Colon carcinoma is one of the serious malignant tumors, and its prevention and treatment have been a hot Research Topic in the medical field. Gao et al. prepared stable SeNP using lentinan (LNT) as a template, and evaluated its inhibitory activity on colon carcinoma. The average particle size of obtained LNT-SeNP was about 59 nm, which was highly stable at 4°C for over 8 weeks, and maintained zero valence, amorphous or spherical structure. LNT stabilized SeNP through hydrogen bond interaction. LNT-SeNP had no significant cytotoxicity on normal cells (IEC-6), but significantly inhibited the proliferation of five types of colon carcinoma cells (HCT-116, HT-29, Caco-2, SW620, and CT26). Among them, LNT-SeNP had the highest sensitivity to HCT-116 cells with an IC_50_ value of 7.65 μM. In addition, LNT-SeNP promoted apoptosis of HCT-116 cells by activating mitochondrial mediated apoptosis pathways. At the same time, LNT-SeNPs induced arrest of development in HCT-116 cells during G0/G1 phase by regulating cell cycle regulatory proteins. These results indicate that LNT-SeNP has a promising application prospect in the treatment of colon carcinoma.

Selenium is capable of regulating autophagy through different signaling pathways, playing an important role in maintaining normal cellular physiological functions and protecting organisms. SeNPs exhibit better biological activity compared to inorganic Se, and research on the regulatory effects of SeNPs on cellular signaling pathways is gradually increasing. Chen D. et al. reviewed the latest research progress on SeNPs inhibiting cancer cell growth or inducing cancer cell death by promoting autophagy in cancer cells. Numerous studies showed that SeNPs enhanced the autophagy ability of cancer cells by activating the ROS mediated JNK pathway and inhibiting the PI3K/Akt/mTOR pathway, achieving better anticancer effects. SeNPs were also used to treat cancer by combination with current chemotherapy methods, achieving better therapeutic effects. In addition, Chen D. et al. summarized the current research advance of SeNPs in reducing drug toxicity, regulating inflammatory response, resisting pathogen infection and treating Alzheimer's disease by regulating autophagy. This review enables us to recognize that SeNPs can serve as a novel autophagy regulator, providing new possibilities for the clinical treatment of some important diseases (Chen D. et al.).

Selenoproteins relates to glucose metabolism or insulin resistance, but the relationship between serum Se level and type 2 diabetes is uncertain. Zhao et al. reviewed the latest research advance in the regulation of Se on metabolism and type 2 diabetes. Results showed that Se supply was very important for maintaining health and glucose and lipid homeostasis in type 2 diabetes patients. Epidemiological analysis of blood Se level in type 2 diabetes patients showed that both Se deficiency and severe excess led to insulin resistance and β cell dysfunction. The potential molecular mechanisms of β cell dysfunction included oxidative stress, insulin signal transduction, interference of gluconeogenesis and endoplasmic reticulum stress. In this regard, Se supplementation requires personalized nutritional recommendations, which needs to consider the regional characteristics and genetic characteristics of individual (Zhao et al.).

In addition to the concentration of Se, the form, valence state and source of Se also have a significant impact on its bioactivity. Tangjaidee et al. introduced the enrichment capacity and forms of Se in Se-rich plant food, the biological effects of Se and Se-containing compounds on human health, and the prospect of Se-rich plant food. The author believed that the biological characteristics of Se-rich plant food were closely related to the chemical form and Se content. Selenium-rich plant food with non-toxic concentration could provide health benefits by increasing the antioxidant activity in human serum. Other biological characteristics, such as antioxidant activity, anti-diabetes and anti-cancer activities, were also demonstrated *in vitro* cell culture models and *in vivo* animal experiments. In contrast, there are still relatively few available data of clinical trials about the effect of Se-rich plant food on human health, thus it is crucial to obtain valuable information supporting the beneficial effect of Se-rich food on human body.

It is an important application of Se to enhance the growth performance, disease resistance and meat quality of poultry through Se supplementation in feed. Xu et al. explored the effects of feeds containing sodium selenite (SeNa), Se yeast (SeY) and Se-rich *Cardamine violifolia* (SeCv) on the growth performance, antioxidant capacity and meat quality of broilers. Results indicated that the broilers fed with SeCv enhanced the average daily gain and ratio of feed to gain compared to those of the broilers fed with SeNa and SeY during the earlier stage (the earlier 21 days). Meanwhile, the total liver anti-oxidative capacity of broilers fed with SeCv was enhanced compared with those of broilers fed with the other two Se sources. In the later stage (from days 21 to 42), the intestinal mucosal morphology of broilers fed with SeCv was improved, and lower liver malondialdehyde contents in the broilers fed with SeCv and SeY were found when compared with that of broilers fed with SeNa. As for the meat quality, SeCv increased the redness of thigh muscle and decreased the cooking loss in both breast and thigh muscle of the boilers compared with those of the broilers fed with SeNa. The above investigation demonstrated that SeCv feed might be a potential Se source to improve the growth performance, antioxidant capacity and meat quality of poultry.

Nano-selenium (nano-Se) was generally regarded as a biostimulant for plants. However, the specific effect on the Chinese herbal medicine was scarcely reported. Dong et al. sprayed nano-Se on the leaves of *Panax notoginseng* (SePN) during its growth. Compared with the control (PN), it was found that spraying nano-Se on the leaves increased the saponin contents (Rb2, Rb3, Rc, F2, Rb2, and Rf) in root of *Panax notoginseng*, of them, a significant increase of 3.9-time of Rb2 content was observed. Animal study showed that the liver malondialdehyde content of mice taking SePN decreased significantly (*p* < 0.01) compared with the control group, and its glutathione content increased significantly (*p* < 0.05). Meanwhile, it increased the enzyme activities involved in glycolipid metabolism (ATGL and PFK), alleviated inflammation and regulated genes related to fatty acid oxidation (MCAD, PPAR-α, and PCSK9) expression of mice. It can be concluded that nano-Se bioaugmentation improved the bioactivity of *Panax notoginseng*.

Soy sauce is rich in free amino acids, low molecular weight peptides and phenolic substances, which have strong antioxidant activity, making it a good carrier of Se. Chen J. et al. added nano-Se during soybean soaking and investigated the bioconversion activity of soybean on Se and the effect of nano-Se on soy sauce quality. Results showed that the total and organic Se contents of soy sauces increased by 32–191 times compared with those of the control, of them, organic Se content accounted for more than 90% of the total Se in soy sauce. Soy sauce prepared by soaking soybeans in 6 mg/L nano-Se solution had the strongest antioxidant activity, which was 9.25–28.02% higher than the control. Nano-Se (6 mg/L) significantly increased the enzyme activities of Daqu (9.76–33.59%), promoted the release of total phenols (27.54%), total flavonoids (27.27%) in soybeans, and the formation of free amino acids (16.19%) and Maillard reaction products (24.50%) in soy sauce. The above method not only significantly increased the organic Se content in soy sauce, but also improved the antioxidant activity of soy sauce, indicating that nano-Se has a broader application prospect in Se-rich fermented foods.

This Research Topic provides valuable information about the latest research on Se and human health. We hope the Research Topic can contribute to scientific advance, more importantly, make people aware of the importance and complexity of the relationship between Se and human health. To the best of our knowledge, the following aspects are the future research hotspots: (1) More extensive epidemiological study on serum Se content and human health, (2) The effect of different valence states of Se (inorganic Se, organic Se and nano-Se) on diseases caused by selenium deficiency and the related mechanism, (3) Development of Se-rich foods, and the effect of Se-rich foods on human health and the related mechanism.

## Author contributions

XG: Data curation, Funding acquisition, Supervision, Validation, Writing—original draft, Writing—review and editing.

## References

[1] Ahmad S Bailey EH Arshad M Ahmed S Watts MJ Stewart AG. Environmental and human iodine and selenium status: lessons from Gilgit-Baltistan, North-East Pakistan. Environ Geochem Health. (2021) 43:4665–86. 10.1007/s10653-021-00943-w 33961155 PMC8528744

[2] WHO. Guidelines for Drinking-Water Quality. Geneva: WHO (2011).

[3] Filippini T Michalke B Wise LA Malagoli C Malavolti M Vescovi L. Diet composition and serum levels of selenium species: a cross-sectional study. Food Chem Toxicol. (2018) 115:482–90. 10.1016/j.fct.2018.03.048 29621579

[4] Zhang J Zhou H Li H Ying Z Liu X. Research progress on separation of selenoproteins/Se-enriched peptides and their physiological activities. Food Funct. (2021) 12:1390–401. 10.1039/D0FO02236E 33464257

[5] Surai PF Fisinin VI. Selenium in pig nutrition and reproduction: boars and semen quality—A review. Asian Aust J Anim Sci. (2015) 28:730–46. 10.5713/ajas.14.0593 25924964 PMC4413004

[6] Tapiero H Townsend DM Tew KD. The antioxidant role of selenium and seleno-compounds. Biomed Pharmacother. (2003) 57:134–44. 10.1016/S0753-3322(03)00035-0 12818475 PMC6361120

[7] Cox DN Bastiaans K. Understanding Australian consumers' perceptions of selenium and motivations to consume selenium enriched foods. Food Qual Prefer. (2007) 18:66–76. 10.1016/j.foodqual.2005.07.015

[8] Gao XL Ye C Ma HL Zhang ZK Wang JC Zhang ZH. Research advances in preparation, stability, application, and possible risks of nanoselenium: Focus on food and food-related fields. J Agric Food Chem. (2023) 71:8731–45. 10.1021/acs.jafc.3c02714 37277939

